# 2-[4-(Azido­meth­yl)phen­yl]benzonitrile

**DOI:** 10.1107/S1600536812029261

**Published:** 2012-06-30

**Authors:** Bo Peng

**Affiliations:** a Department of Applied Chemistry, Nanjing College of Chemical Technology, Nanjing 210048, People’s Republic of China

## Abstract

The title compound, C_14_H_10_N_4_, was obtained by a reaction of 4′-(bromo­meth­yl)biphenyl-2-carbonitrile and sodium azide. The dihedral angle between the benzene rings is 46.41 (7)°. Weak inter­molecular C—H⋯π inter­actions occur in the crystal.

## Related literature

For background literature, see: Haertling (1999 ▶); Homes *et al.* (2001 ▶).
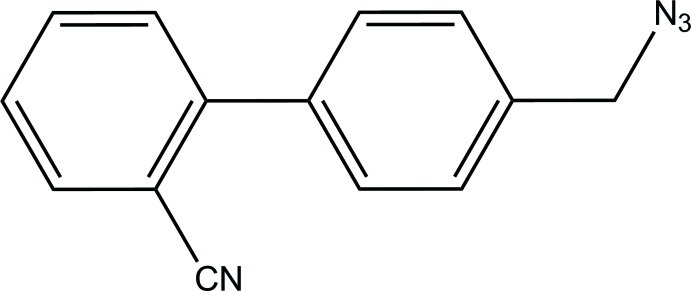


## Experimental

### 

#### Crystal data

C_14_H_10_N_4_
*M* *_r_* = 234.26
Triclinic, 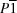

*a* = 8.0763 (16) Å
*b* = 8.2183 (16) Å
*c* = 10.116 (2) Å
α = 76.22 (3)°
β = 69.36 (3)°
γ = 85.94 (3)°
*V* = 610.2 (2) Å^3^
*Z* = 2
Mo *K*α radiation
μ = 0.08 mm^−1^
*T* = 293 K
0.20 × 0.20 × 0.20 mm

#### Data collection

Rigaku Mercury2 diffractometer
6204 measured reflections
2748 independent reflections
1559 reflections with *I* > 2σ(*I*)
*R*_int_ = 0.035

#### Refinement

*R*[*F* ^2^ > 2σ(*F* ^2^)] = 0.058
*wR*(*F* ^2^) = 0.146
*S* = 1.02
2748 reflections
163 parameters
H-atom parameters constrained
Δρ_max_ = 0.14 e Å^−3^
Δρ_min_ = −0.21 e Å^−3^

### 

Data collection: *CrystalClear* (Rigaku, 2005 ▶); cell refinement: *CrystalClear*; data reduction: *CrystalClear*; program(s) used to solve structure: *SHELXS97* (Sheldrick, 2008 ▶); program(s) used to refine structure: *SHELXL97* (Sheldrick, 2008 ▶); molecular graphics: *SHELXTL* (Sheldrick, 2008 ▶); software used to prepare material for publication: *SHELXTL*.

## Supplementary Material

Crystal structure: contains datablock(s) I, global. DOI: 10.1107/S1600536812029261/xu5575sup1.cif

Structure factors: contains datablock(s) I. DOI: 10.1107/S1600536812029261/xu5575Isup2.hkl

Supplementary material file. DOI: 10.1107/S1600536812029261/xu5575Isup3.cml

Additional supplementary materials:  crystallographic information; 3D view; checkCIF report

## Figures and Tables

**Table 1:** Hydrogen-bond geometry (Å, °) *Cg* is the centroid of the C7–C12 benzene ring.

*D*—H⋯*A*	*D*—H	H⋯*A*	*D*⋯*A*	*D*—H⋯*A*
C14—H14*B*⋯*Cg* ^i^	0.97	2.75	3.642 (3)	154

Symmetry code: (i) 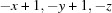.

**Fig. 1.**
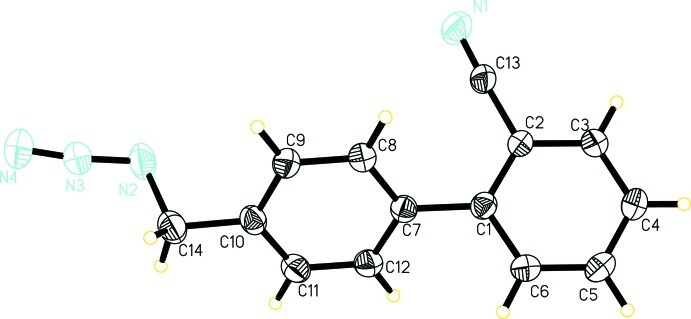
Perspective structure of the title compound, showing the atom-numbering scheme. Displacement ellipsoids are drawn at the 30% probability level.

## supplementary crystallographic information

### Comment

At present, much attention in ferroelectric material field is focused on developing ferroelectric pure organic or inorganic compounds (Haertling *et al.* 1999; Homes *et al.* 2001). In order to find more dielectric ferroelectric materials, we investigate the physical properties of the title compound(Fig. 1). The dielectric constant of the title compound as a function of temperature indicates that the permittivity is basically temperature-independent (dielectric constant equaling to 3.5 to 4.8), suggesting that this compound should be not a real ferroelectrics or there may be no distinct phase transition occurred within the measured temperature range. Similarly, below the melting point (373 K) of the compound, the dielectric constant as a function of temperature also goes smoothly, and there is no dielectric anomaly observed (dielectric constant equaling to 3.5 to 4.8).Herein, we report the synthesis and crystal structure of the title compound, 2-[4-(azidomethyl)phenyl]benzonitrile.

Molecules of the title compound have normal geometric parameters. The bond lengths and angles are within their normal ranges. All benzene rings are planar and the azide group is linear.The dihedral angle between the benzene rings in the molecule is 46.41 (7). Dipole–dipole and van der Waals interactions are effective in the molecular packing.

### Experimental

To a stirred solution of 4'-(bromomethyl)biphenyl-2-carbonitrile (5.42 g, 0.02 mol) in 30 mL of methanol, sodium azide (1.3 g, 0.02 mol) was added at the room temperature. The temperature was raised to 50°C in half an hour gradually and the mixture was stirred at this temperature for 12 h. The precipitate was filtered and washed with a small amount of water. The title compound was isolated using column chromatography (Petroleum ether: ethyl acetate-4:1). Single crystals suitable for X-ray diffraction analysis were obtained

### Refinement

The H-atoms bonded to the C-atom were positioned geometrically and refined using a riding model, with C—H = 0.93 Å and *U*_iso_(H) = 1.2*U*_eq_(C).

### Crystal data

**Table:**

C_14_H_10_N_4_	*Z* = 2
*M**_r_* = 234.26	*F*(000) = 244
Triclinic, *P*1	*D*_x_ = 1.275 Mg m^−^^3^
Hall symbol: -P 1	Mo *K*α radiation, λ = 0.71073 Å
*a* = 8.0763 (16) Å	Cell parameters from 2748 reflections
*b* = 8.2183 (16) Å	θ = 2.6–27.4°
*c* = 10.116 (2) Å	µ = 0.08 mm^−^^1^
α = 76.22 (3)°	*T* = 293 K
β = 69.36 (3)°	Prism, colorless
γ = 85.94 (3)°	0.20 × 0.20 × 0.20 mm
*V* = 610.2 (2) Å^3^	

### Data collection

**Table:**

Rigaku Mercury2 diffractometer	1559 reflections with *I* > 2σ(*I*)
Radiation source: fine-focus sealed tube	*R*_int_ = 0.035
Graphite monochromator	θ_max_ = 27.4°, θ_min_ = 3.5°
Detector resolution: 13.6612 pixels mm^-1^	*h* = −10→10
CCD_Profile_fitting scans	*k* = −10→10
6204 measured reflections	*l* = −13→13
2748 independent reflections	

### Refinement

**Table:**

Refinement on *F*^2^	Primary atom site location: structure-invariant direct methods
Least-squares matrix: full	Secondary atom site location: difference Fourier map
*R*[*F*^2^ > 2σ(*F*^2^)] = 0.058	Hydrogen site location: inferred from neighbouring sites
*wR*(*F*^2^) = 0.146	H-atom parameters constrained
*S* = 1.02	*w* = 1/[σ^2^(*F*_o_^2^) + (0.0613*P*)^2^ + 0.0517*P*] where *P* = (*F*_o_^2^ + 2*F*_c_^2^)/3
2748 reflections	(Δ/σ)_max_ = 0.001
163 parameters	Δρ_max_ = 0.14 e Å^−^^3^
0 restraints	Δρ_min_ = −0.21 e Å^−^^3^

### Special details

**Table:**

Geometry. All e.s.d.'s (except the e.s.d. in the dihedral angle between two l.s. planes) are estimated using the full covariance matrix. The cell e.s.d.'s are taken into account individually in the estimation of e.s.d.'s in distances, angles and torsion angles; correlations between e.s.d.'s in cell parameters are only used when they are defined by crystal symmetry. An approximate (isotropic) treatment of cell e.s.d.'s is used for estimating e.s.d.'s involving l.s. planes.
Refinement. Refinement of *F*^2^ against ALL reflections. The weighted *R*-factor *wR* and goodness of fit *S* are based on *F*^2^, conventional *R*-factors *R* are based on *F*, with *F* set to zero for negative *F*^2^. The threshold expression of *F*^2^ > σ(*F*^2^) is used only for calculating *R*-factors(gt) *etc*. and is not relevant to the choice of reflections for refinement. *R*-factors based on *F*^2^ are statistically about twice as large as those based on *F*, and *R*- factors based on ALL data will be even larger.

### Fractional atomic coordinates and isotropic or equivalent isotropic displacement parameters (Å^2^)

**Table:**

	*x*	*y*	*z*	*U*_iso_*/*U*_eq_	
C7	0.0733 (2)	0.6414 (2)	0.1795 (2)	0.0441 (5)	
C1	−0.0353 (2)	0.7492 (2)	0.2778 (2)	0.0444 (5)	
C9	0.2358 (3)	0.3876 (2)	0.1389 (2)	0.0519 (5)	
H9A	0.2727	0.2827	0.1769	0.062*	
C11	0.2308 (3)	0.6036 (3)	−0.0645 (2)	0.0565 (5)	
H11A	0.2629	0.6449	−0.1643	0.068*	
C8	0.1297 (2)	0.4830 (2)	0.2324 (2)	0.0482 (5)	
H8A	0.0957	0.4404	0.3321	0.058*	
C3	−0.2866 (3)	0.7958 (2)	0.4875 (2)	0.0545 (5)	
H3A	−0.3858	0.7539	0.5675	0.065*	
C12	0.1262 (3)	0.7001 (2)	0.0292 (2)	0.0538 (5)	
H12A	0.0908	0.8055	−0.0091	0.065*	
C6	0.0093 (3)	0.9192 (2)	0.2491 (2)	0.0579 (6)	
H6A	0.1082	0.9631	0.1695	0.070*	
C2	−0.1865 (2)	0.6899 (2)	0.3994 (2)	0.0446 (5)	
C14	0.4070 (3)	0.3449 (3)	−0.1131 (2)	0.0662 (6)	
H14A	0.4233	0.4016	−0.2127	0.079*	
H14B	0.5220	0.3337	−0.1020	0.079*	
C10	0.2877 (2)	0.4470 (2)	−0.0112 (2)	0.0499 (5)	
C13	−0.2470 (3)	0.5166 (3)	0.4378 (2)	0.0555 (5)	
C5	−0.0900 (3)	1.0228 (3)	0.3361 (2)	0.0655 (6)	
H5A	−0.0571	1.1349	0.3142	0.079*	
C4	−0.2381 (3)	0.9620 (2)	0.4556 (2)	0.0612 (6)	
H4A	−0.3041	1.0327	0.5137	0.073*	
N1	−0.2994 (3)	0.3814 (2)	0.4726 (2)	0.0849 (7)	
N3	0.4183 (2)	0.0789 (2)	−0.14723 (19)	0.0640 (5)	
N2	0.3271 (3)	0.1777 (2)	−0.0802 (2)	0.0799 (6)	
N4	0.4870 (3)	−0.0238 (3)	−0.2027 (3)	0.1025 (8)	

### Atomic displacement parameters (Å^2^)

**Table:**

	*U*^11^	*U*^22^	*U*^33^	*U*^12^	*U*^13^	*U*^23^
C7	0.0391 (10)	0.0458 (11)	0.0475 (11)	0.0000 (8)	−0.0138 (9)	−0.0123 (9)
C1	0.0476 (11)	0.0399 (10)	0.0465 (11)	0.0041 (9)	−0.0176 (9)	−0.0100 (9)
C9	0.0494 (11)	0.0464 (11)	0.0566 (13)	0.0062 (9)	−0.0146 (10)	−0.0128 (10)
C11	0.0573 (12)	0.0613 (13)	0.0447 (11)	−0.0030 (11)	−0.0098 (10)	−0.0113 (10)
C8	0.0487 (11)	0.0478 (11)	0.0465 (11)	0.0024 (9)	−0.0139 (10)	−0.0123 (9)
C3	0.0575 (12)	0.0490 (12)	0.0486 (12)	0.0016 (10)	−0.0079 (10)	−0.0120 (10)
C12	0.0551 (12)	0.0487 (11)	0.0511 (12)	0.0022 (10)	−0.0140 (10)	−0.0062 (10)
C6	0.0596 (13)	0.0462 (11)	0.0567 (13)	−0.0065 (10)	−0.0073 (11)	−0.0084 (10)
C2	0.0479 (11)	0.0382 (10)	0.0467 (11)	0.0005 (9)	−0.0140 (9)	−0.0114 (9)
C14	0.0547 (13)	0.0708 (15)	0.0653 (15)	−0.0037 (12)	−0.0034 (11)	−0.0266 (12)
C10	0.0395 (10)	0.0560 (12)	0.0525 (12)	−0.0037 (9)	−0.0080 (9)	−0.0202 (10)
C13	0.0507 (12)	0.0486 (12)	0.0593 (13)	0.0009 (10)	−0.0055 (10)	−0.0185 (11)
C5	0.0763 (16)	0.0402 (11)	0.0744 (16)	−0.0047 (11)	−0.0167 (13)	−0.0153 (11)
C4	0.0704 (14)	0.0466 (12)	0.0632 (14)	0.0088 (11)	−0.0148 (12)	−0.0212 (11)
N1	0.0769 (14)	0.0530 (12)	0.1028 (17)	−0.0111 (11)	0.0031 (12)	−0.0261 (12)
N3	0.0634 (12)	0.0643 (12)	0.0597 (12)	0.0051 (10)	−0.0131 (10)	−0.0197 (10)
N2	0.0686 (12)	0.0743 (13)	0.0863 (14)	−0.0054 (11)	0.0052 (11)	−0.0461 (12)
N4	0.1053 (18)	0.0736 (15)	0.1081 (19)	0.0189 (14)	−0.0048 (15)	−0.0375 (14)

### Geometric parameters (Å, º)

**Table:**

C7—C8	1.395 (2)	C12—H12A	0.9300
C7—C12	1.397 (3)	C6—C5	1.381 (3)
C7—C1	1.496 (2)	C6—H6A	0.9300
C1—C6	1.403 (2)	C2—C13	1.456 (3)
C1—C2	1.408 (3)	C14—N2	1.474 (3)
C9—C8	1.389 (2)	C14—C10	1.514 (3)
C9—C10	1.395 (3)	C14—H14A	0.9700
C9—H9A	0.9300	C14—H14B	0.9700
C11—C10	1.386 (3)	C13—N1	1.146 (2)
C11—C12	1.393 (3)	C5—C4	1.385 (3)
C11—H11A	0.9300	C5—H5A	0.9300
C8—H8A	0.9300	C4—H4A	0.9300
C3—C4	1.378 (3)	N3—N4	1.128 (2)
C3—C2	1.403 (2)	N3—N2	1.220 (2)
C3—H3A	0.9300		
			
C8—C7—C12	117.61 (17)	C1—C6—H6A	119.2
C8—C7—C1	122.25 (17)	C3—C2—C1	121.39 (17)
C12—C7—C1	120.11 (17)	C3—C2—C13	117.21 (17)
C6—C1—C2	116.68 (17)	C1—C2—C13	121.39 (16)
C6—C1—C7	120.17 (17)	N2—C14—C10	109.67 (17)
C2—C1—C7	123.13 (16)	N2—C14—H14A	109.7
C8—C9—C10	120.86 (18)	C10—C14—H14A	109.7
C8—C9—H9A	119.6	N2—C14—H14B	109.7
C10—C9—H9A	119.6	C10—C14—H14B	109.7
C10—C11—C12	120.88 (19)	H14A—C14—H14B	108.2
C10—C11—H11A	119.6	C11—C10—C9	118.27 (17)
C12—C11—H11A	119.6	C11—C10—C14	120.86 (19)
C9—C8—C7	121.18 (18)	C9—C10—C14	120.84 (19)
C9—C8—H8A	119.4	N1—C13—C2	177.4 (2)
C7—C8—H8A	119.4	C6—C5—C4	120.90 (19)
C4—C3—C2	120.07 (19)	C6—C5—H5A	119.6
C4—C3—H3A	120.0	C4—C5—H5A	119.6
C2—C3—H3A	120.0	C3—C4—C5	119.36 (19)
C11—C12—C7	121.20 (19)	C3—C4—H4A	120.3
C11—C12—H12A	119.4	C5—C4—H4A	120.3
C7—C12—H12A	119.4	N4—N3—N2	172.5 (2)
C5—C6—C1	121.59 (19)	N3—N2—C14	115.65 (18)
C5—C6—H6A	119.2		

### Hydrogen-bond geometry (Å, º)

Cg is the centroid of the C7–C12 benzene ring.

**Table:**

*D*—H···*A*	*D*—H	H···*A*	*D*···*A*	*D*—H···*A*
C14—H14*B*···*Cg*^i^	0.97	2.75	3.642 (3)	154

Symmetry code: (i) −*x*+1, −*y*+1, −*z*.

## References

[bb1] Haertling, G. H. (1999). *J. Am. Ceram. Soc.* **82**, 797–810.

[bb2] Homes, C. C., Vogt, T., Shapiro, S. M., Wakimoto, S. & Ramirez, A. P. (2001). *Science*, **293**, 673–676. 10.1126/science.1061655 11474105

[bb3] Rigaku (2005). *CrystalClear* Rigaku Corporation, Tokyo, Japan.

[bb4] Sheldrick, G. M. (2008). *Acta Cryst.* A**64**, 112–122. 10.1107/S0108767307043930 18156677

